# An Assessment of Three Carbohydrate Metrics of Nutritional Quality for Packaged Foods and Beverages in Australia and Southeast Asia

**DOI:** 10.3390/nu12092771

**Published:** 2020-09-11

**Authors:** Denise Tan, Andrea Nicole Olden, Audrey Orengo, Célia Francey, Vanessa Caroline Campos, Flavia Fayet-Moore, Jung Eun Kim, Kim-Anne Lê

**Affiliations:** 1 Department of Food Science and Technology, National University of Singapore, Singapore 117543, Singapore; Denise.Tan@rdsg.nestle.com; 2 Science and Technology Department, Nestlé R&D Center (Pte) Ltd., Singapore 618802, Singapore; Andrea.Olden@rd.nestle.com; 3 Department of Metabolic Health, Nestlé Research, 1000 Lausanne 26, Switzerland; Audrey.Orengo@rdls.nestle.com (A.O.); Celia.Francey@rd.nestle.com (C.F.); VanessaCaroline.Campos@rdls.nestle.com (V.C.C.); 4 Nutrition Research Australia, Sydney NSW 2000, Australia; flavia@nraus.com

**Keywords:** carbohydrate quality, nutritional quality, nutrient profiling model, free sugars, dietary fibre, packaged foods, Asia Pacific

## Abstract

Carbohydrate quality is an aetiological factor of diet-related disease. Indices of carbohydrate quality featuring various ratios of carbohydrates-to-dietary fibre-to-sugar have been associated with improved product and/or diet quality in westernised countries. Carbohydrate intake is especially high in Asia Pacific. Thus, this study evaluated the ability of such carbohydrate metrics to discriminate the nutritional quality of carbohydrate-rich packaged foods and beverages in Australia, Malaysia, Singapore, Thailand and the Philippines, with an additional focus on beverages. This evaluation was conducted by comparing product nutritional composition and assessing products against three national nutrient profiling models. Results showed that Australia had the highest proportion of products meeting all metrics, compared to the Southeast Asian countries. Beverages had a low adherence to all metrics compared to solid foods. Across the five countries, both processed food and beverages meeting the metrics generally contained higher dietary fibre, protein, and certain vitamins and minerals whilst having lower energy, total sugars, free sugars, trans fat and cholesterol content compared to products not meeting the metrics. The metrics were also aligned with national nutrient profiling models to identify nutritious products. In conclusion, these metrics allowed us to discriminate product nutritional quality in the countries assessed and are applicable to beverages.

## 1. Introduction

Carbohydrates are the primary energy source of the human diet. Its intake is especially high in Asian countries, where the average consumption ranges from 65% to >80% of daily energy intake, contributed primarily by starch-based foods (e.g., rice-based foods) [1,2,3]. In contrast, western countries, such as Australia, have a lower carbohydrate consumption at an average of <55% to 65% of daily energy intake, and consists of a mix of starch-based foods (e.g., breads) and sugars [1,3,4,5].

Recent scientific literature has established that carbohydrate quality over quantity is more predictive of disease risk, and the topic of how to define carbohydrate quality has raised much debate over the past years in an attempt to unify recommendations beyond single nutrient targets, such as “high in fibre” or “low in sugar” [6,7,8,9]. Among the most well-known measures, the glycaemic index (GI) and whole grain content have been proposed as markers of carbohydrate quality in a product. While GI is a notable measure of dietary carbohydrate quality [10,11,12], its use and understanding by the broader population is limited, owing to the measure being too technical and its need to be tested clinically [13]. Whole grain, in contrast, is more broadly known and recognised [14,15,16], but the lack of universal definition of what constitutes a “whole grain food” and a minimum nutritional requirement have led to heterogeneous quality of products bearing a whole grain claim [17,18]. Thus, there is a need to develop a measure of nutritional quality for carbohydrate-rich products, which is nutritionally credible yet straightforward to communicate and compute.

As there is a general consensus that dietary fibre intake, in particular from whole grain sources, should be promoted [6,7,19,20], and free sugars intake be limited [21,22], novel alternative metrics of carbohydrate quality beyond absolute recommendations of these nutrients have emerged. These are expressed as ratios of total carbohydrates and/or starch to dietary fibre. AlEssa and team reported that diets with low total carbohydrates-to-cereal fibre ratios are associated with lower risk for type 2 diabetes mellitus (T2DM) and coronary heart disease in the United States of America (USA) [6,19]. Recently, Blumfield et al., 2020, showed that diets compliant with similar carbohydrate metrics but integrating a free sugar threshold had an improved overall nutritional intake in the Australian population, reflected by a higher Healthy Eating Index score [23]. 

While the relevance of such metrics has been demonstrated at diet level, it remains a challenge for consumers to relate their daily food selections with absolute nutrient recommendations. Packaged foods and beverages make up a large part of the modern-day urban diet, and the ability to select healthful products is a key contribution to healthier diets [24,25,26]. Recently, such metrics have also been proven useful at a product level, with products meeting these metrics found to be nutritionally superior in the USA [17,27] and Brazil [28]. Though the metric has been established in these countries, differences in eating culture and food regulation have a vast influence on the availability and choice of products. For instance, there is a greater history and more extensive range of plant-based milk substitutes in Asia [29,30], and cereal-based drinks, which are consumed extensively as a snack and/or breakfast, are non-negligible sources of carbohydrate intake in these countries [31]. However, due to the low availability of such products in the USA and Brazil, previous studies [27,28] failed to capture whether the carbohydrate metrics could be relevant to beverages. Nutrient labelling and permitted product claims also influence product formulations in the market significantly. For example, unlike the USA, labelling of added and/or free sugars is voluntary in the countries assessed in this paper, and consumer knowledge of its importance may be limited. Thus, whether the metrics developed based on USA-style dietary patterns may apply to products in Asia Pacific, and especially beverages, remains unknown. 

The objective of this study was to investigate whether these carbohydrate metrics could help identify products, particularly beverages, of a higher nutritional quality in countries from the Asia Pacific region, namely Australia, Malaysia, Singapore, Thailand and the Philippines. 

## 2. Materials and Methods

### 2.1. Definition of the Carbohydrate Metrics

Three carbohydrate metrics were assessed; these are, per 10 g of total carbohydrates in a product:

1. At least 1 g of dietary fibre (simple ratio);
2. At least 1 g of dietary fibre and no more than 2 g of free sugars (modified ratio);
3. At least 1 g of dietary fibre, and no more than 2 g of free sugar per 1 g of dietary fibre (dual ratio).

The simple ratio was developed by the American Heart Association (AHA) and follows a recommendation based on the ratio of total carbohydrates to dietary fibre in whole wheat [32]. The modified ratio includes an upper limit for free sugars based on the WHO free sugars recommendations described above [33], and on an average recommendation that about 50% of total energy intake is derived from carbohydrates, then individuals should consume no more than 2 g of free sugar per 10 g of carbohydrates. The dual ratio was developed to put emphasis on the dietary fibre content, rather than the total carbohydrates. In this metric, free sugar content is restricted to 2 g for every 1 g of dietary fibre in the product [27]. This is based on AHA recommendations to consume at least 25 g of dietary fibre [14] and the WHO free sugar recommendation (equivalent to 50 g of free sugars for a diet of 2000 kcal a day). The dual and modified ratio are subsets of the simple ratio and take free sugars into account. 

### 2.2. Food Databases and Product Selection Criteria

Carbohydrate-based packaged foods and beverages from two different databases—Australian Food Composition Database Release 1 (AFCD-1) and Mintel Global New Products Database (Mintel Database)—were curated. Data from AFCD-1 are available as food and beverage sub-categories, whereas data from the Mintel databases are of individual products. AFCD-1 is a national database that contains the nutrient composition of common Australian food and beverage sub-categories [34]. Data were obtained predominantly from analysis of typical Australian products within a sub-category, and the remainder were derived from product labels, imputations or burrowed from other countries. The Mintel Database is a collation of products launched in a country, with details of its on-pack product information including its declared nutrient content, product description, ingredients list and claims [35]. To ensure identification of the most relevant products—predominantly cereal-based—packaged foods and beverages with more than 50% energy from carbohydrates were included in the analysis.

A total of 127 carbohydrate-based packaged food and beverage sub-categories in AFCD-1 were assessed. Several sub-categories in the database were similar in sampling and nutritional composition (e.g., “coconut, fresh, mature, water or juice” and “coconut, fresh, young or immature, water or juice”) and were merged, resulting in a revised total of 101 sub-categories, including 86 food sub-categories and 15 beverage sub-categories.

A total of 8390 carbohydrate-based packaged food and beverage products in the Mintel Database were assessed. These included products from Australia (Mintel Australia) and four Southeast Asian countries (Mintel Asia) during the period of January 2014–August 2019. Mintel Asia consisted of products from Malaysia, Singapore, Thailand and the Philippines. 

All sub-categories from AFCD-1 and products from the Mintel Database assessed were classified into 12 food categories and 6 beverage categories (Table 1). Analyses on beverages were separated into ready-to-drink (RTD) and powdered beverages due to differences in nutrient declaration between the two categories (i.e., “per 100 g” for powdered beverages and “per 100 mL” for RTD beverages).

### 2.3. Proportion of Carbohydrate-Based Packaged Foods and Beverages Based on the Carbohydrate Metrics

The sub-categories from AFCD-1 were weighted by the number of products available on the market for each category using the Mintel Database and then assessed for whether they passed each of the three carbohydrate metrics.

**Figure 1:**
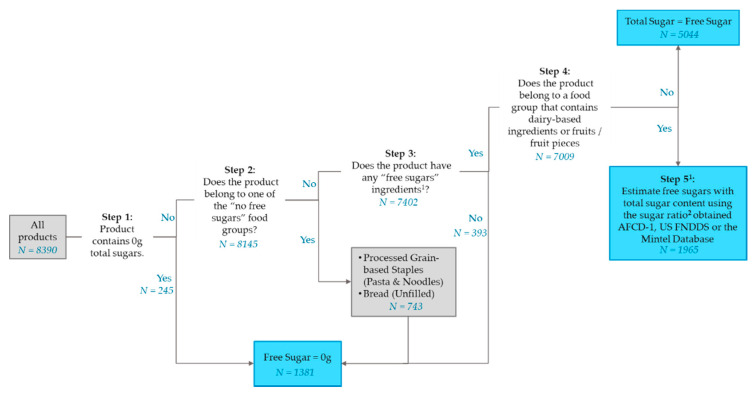
Free sugars imputation process for determining the free sugars content of products from the MintelDatabase. ^1^ “Free sugars” ingredients refers to sugar, maltodextrin, syrup, sucrose, glucose, fructose, maltose, honey, nectar, malt, non-dairy creamer, juice, jam, sweetened ingredients (e.g., sweetened fruits). ^2^ Sugar ratio refers to the ratio of free sugars:total sugars. This is the mean content of free sugars divided by the mean total sugars content of a product sub-category with dairy-based ingredients or whole fruit/fruit pieces. These were obtained from either the Australian Food Composition Database Release 1 (AFCD-1), US Food and Nutrient Database for Dietary Studies (FNDDS) or declared values from the Mintel Database.

The products from the Mintel Database were evaluated for their ability to meet each metric using their declared carbohydrates, dietary fibre and sugar content. As it is not mandatory for free sugars to be declared on packaging in these countries, the former was obtained through an imputation from the product’s total sugars content using a modified methodology adopted from Louie et al., 2015 [36] (Figure 1). 

A product category was considered to have a high adherence to a metric if at least 40% of products in the category met its criteria (highest quartile of product categories), and moderate adherence to the metric if at least 20% but less than 40% of products in the category met its criteria. A product category was considered to have a low adherence to a metric if less than 20% of products met the metric. 

### 2.4. Nutritional Quality of Carbohydrate-Based Packaged Foods and Beverages Based on the Carbohydrate Metrics

#### 2.4.1. Evaluation of Product Nutritional Composition

The nutritional quality between foods and beverages that passed and failed each metric was first evaluated by comparing the level of nutrients. 

For AFCD-1, this was carried out by using the energy, protein, total fat, saturated fat, trans fat, cholesterol, total sugar, free sugar, dietary fibre, sodium, calcium, potassium, iron, iodine, magnesium, zinc, selenium and vitamins A (retinol equivalent), E, B1, B2, B3, B6, B9 and B12 values of sub-categories from the database.

For the Mintel Database, the nutritional comparison between products that passed or failed each metric was assessed by using the declared energy, carbohydrates, protein, total fat, saturated fat, total sugar, dietary fibre and sodium content. An additional category-specific analysis was performed on categories that had the highest adherence to the metrics—hot cereals, cold cereals, cereal and fruit bars and breads (unfilled).

Due to large variations in serving size across the range of sub-categories and products assessed, all nutrient comparisons were conducted as per 100 g/100 mL of product. 

#### 2.4.2. Evaluation of Products against Nutrient Profiling Models

In addition to comparing individual nutrients, products from the Mintel Database were also evaluated against national nutrient profiling models specific to the region. Products from Mintel Australia were evaluated against the Food Standards Australia New Zealand Nutrient Profiling Score Criterion (NPSC) and the Australia New Zealand Health Star Rating (HSR). Products from Mintel Asia were assessed against NPSC, HSR and the Singapore Healthier Choice Symbol (HCS). Under the guidelines of the respective nutrient profiling models, a product is considered a nutritious product if it is within the category-specific score limit for the NPSC, ≥3.5 stars for the HSR or passed the HCS [37,38,39]. The fruits, vegetable, nut and legume content and whole grain content of products were required for this assessment; where undeclared, these values were imputed to complete this assessment.

This evaluation was not carried out for the AFCD-1 as the database is comprised of the nutrient composition of sub-categories and not individual products.

### 2.5. Proportion of Whole Grain Product Choices and Their Association with the Carbohydrate Metrics

As each carbohydrate metric stemmed from the carbohydrates and dietary fibre content of whole grains, the number of whole grain product choices from the Mintel Database (i.e., products that had whole grain claims or communication) were quantified. To determine if this was associated with the metrics, this parameter was compared with products that passed and failed each metric.

### 2.6. Comparison of Data from AFCD-1 and Mintel Australia

A qualitative comparison of product categories that met each metric from the two Australian databases (AFCD-1 and Mintel Australia) was carried out. 

The imputed free sugars content used in the Mintel Database was compared against the free sugars content of sub-categories from the same product category in AFCD-1. 

### 2.7. Statistical Analysis

Unequal variances *t*-tests were performed to analyse differences in nutritional composition between product sub-categories from AFCD-1 and products from the Mintel Database that passed and failed the metrics.

The Pearson’s chi-square test was conducted to determine significant associations between the carbohydrate metrics and whole grain product variants amongst products from the Mintel Database.

To validate that the free sugars estimation calculated for Mintel database using the modified Louie methodology was similar to that of AFCD-1, a paired two-tailed t-test was performed and the Nash–Sutcliffe efficiency (NSE) model coefficient was determined.

All statistical analyses were performed using Mintab 18 and Microsoft Excel 2016, and *p* < 0.05 was used as the criterion to determine statistical significance.

## 3. Results

### 3.1. Proportion of Carbohydrate-Based Packaged Foods and Beverages Based on the Carbohydrate Metrics

**Figure 2:**
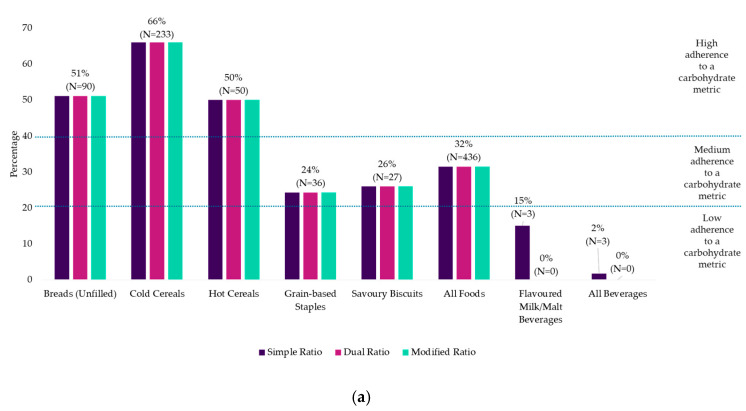

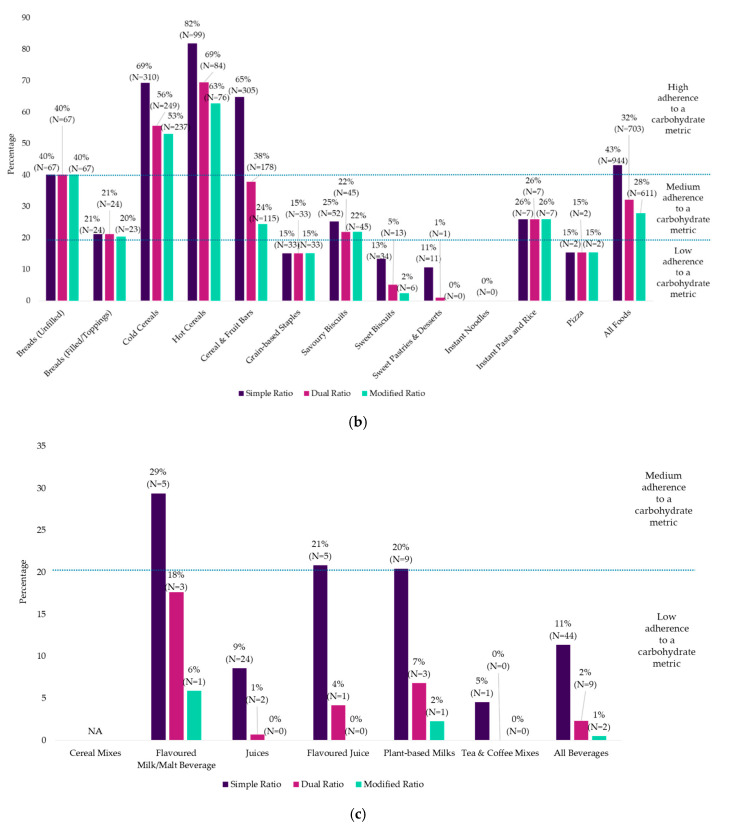

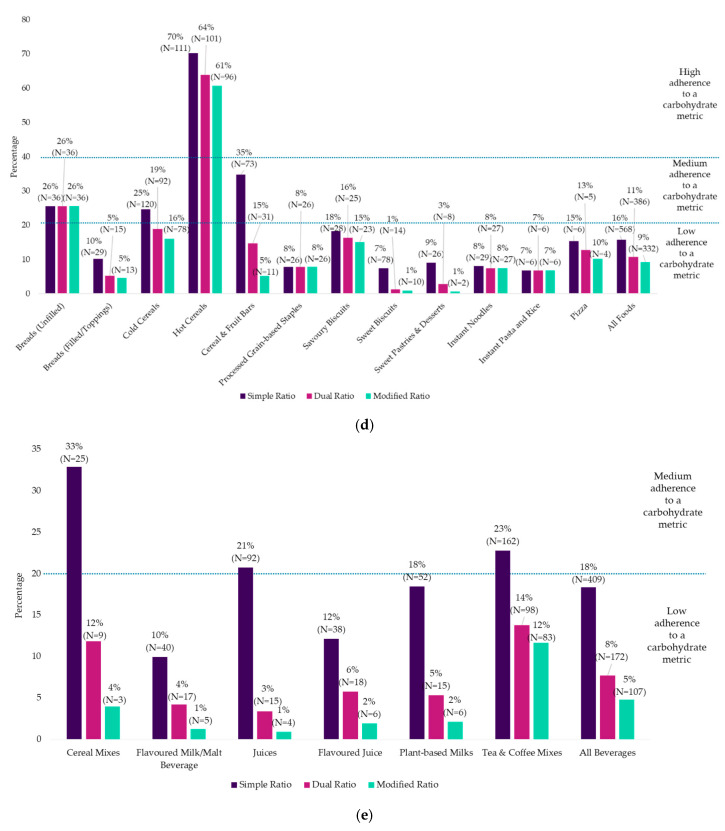
Percentage of carbohydrate-based packaged food and beverage sub-categories and products from AFCD-1 and the Mintel Database that met each carbohydrate metric. (**a**) Comparison of carbohydrate-based packaged food and beverage sub-categories from AFCD-1. The following categories were omitted from this graph as no sub-categories passed any metric: breads (filled/toppings), cereal and fruit bars, sweet biscuits, sweet pastries and desserts, instant noodles, instant pasta and rice, pizza, cereal mixes, juices, flavoured juice, plant-based milks and tea and coffee mixes. (**b**) Comparison of carbohydrate-based packaged foods from Mintel Australia. (**c**) Comparison of carbohydrate-based packaged beverages from Mintel Australia. NA indicates no products within the category from the respective database. (**d**) Comparison of carbohydrate-based packaged foods from Mintel Asia. (**e**) Comparison of carbohydrate-based packaged beverages from Mintel Asia.

In the AFCD-1, all food sub-categories that passed the simple ratio (32%) also passed the dual and modified ratios, whilst only one beverage sub-category passed the simple ratio (2%) and no beverage sub-categories passed the dual or modified ratio (Figure 2a). 

When carbohydrate-based products from Mintel Australia were assessed, 43%, 32% and 28% of foods met the simple, dual and modified ratios, respectively, whilst only 11%, 2% and 1% of beverages met each of these metrics (Figure 2b,c). In Mintel Asia, 16%, 11% and 9% of foods and 18%, 8% and 5% of beverages met the simple ratio, dual ratio and modified ratio, respectively (Figure 2d,e).

From the Mintel Database, foods that were predominantly cereal-based had the highest adherence to all metrics, in particular, hot cereals, cold cereals, breads (unfilled) and cereal and fruit bars. None of the beverage categories had a high adherence to any metric, though various beverage categories had a moderate adherence to the simple ratio in selected countries (Figure 2c,e). There was a considerable drop in adherence to the metrics when free sugars were taken into consideration (i.e., dual and modified ratios) and this was primarily due to the major decline in the proportion of cereal and fruit bars and beverages meeting these metrics. However, cereal and fruit bars still had a moderate adherence to the dual ratio (38%) and modified ratio (24%) in Australia. When free sugars were taken into account, then all beverage categories had a low adherence to any metric in any database.

**Figure 3:**
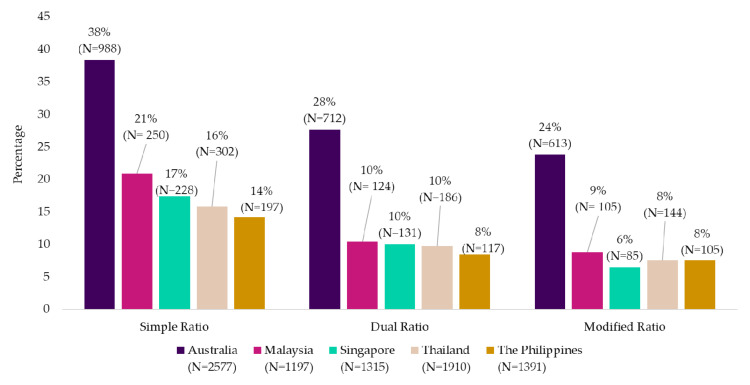
Comparison of the percentage of carbohydrate-based packaged foods and beverages from the Mintel Database that meet each metric across the five countries (*n* = 8390).

When comparing the range products from the Mintel Database assessed across the five countries, Australia was found to have the largest proportion of products that met the metrics (approximately two times more) compared to Southeast Asian countries (Figure 3).

### 3.2. Nutritional Quality of Carbohydrate-Based Packaged Foods and Beverages Based on the Carbohydrate Metrics

#### 3.2.1. Comparison of the Nutritional Composition between Carbohydrate-Based Packaged Foods

From the assessment of products from AFCD-1, foods that met all metrics had significantly higher levels of dietary fibre (+264%), protein (+34%), iron (+122%), magnesium (+220%), potassium (+101%), zinc (+106%), selenium (+43%), vitamins B1 (+152%), B3 (+137%) and B9 (+111%) and lower levels of total sugars (−58%), free sugars (−78%) saturated fat (−76%), trans fat (−88%) and cholesterol (−100%) content compared to foods that did not meet any metric (Table 2). However, these products also had significantly lower levels of Vitamin A (−98%) and B12 (−91%).

Foods from Mintel Australia that met the dual and modified ratios were found to have a significantly greater fibre (+167% and +124%, respectively) and protein content (+35% and +30%, respectively) and a lower total energy (−4% and −6%, respectively), total sugars (−23% and −26%, respectively), free sugars (−65% and −80%, respectively) and saturated fat (−34% and −40%, respectively) content compared to foods that did not pass any metric (Table 3).

In the analysis of products from Mintel Asia, foods that passed the simple, dual and modified ratios all had a higher dietary fibre (+246%, +236% and +211%, respectively) and protein content (+27%, +37% and +40%, respectively), in addition to a reduced energy (−7%, −11% and −11%, respectively), total sugar (−33%, −60% and −68%, respectively), free sugar (−43%, −76% and −87%, respectively), total fat (−14%, −28% and −29%, respectively), saturated fat (−34%, −53% and −54%, respectively) and sodium (−76%, −75% and −73%, respectively) content compared to foods that did not pass the ratio (Table 4). 

When a category-specific analysis of hot cereals, cold cereals, cereal and fruit bars and breads (unfilled) was performed, products that met all the ratios had better levels of positive nutrients and lower levels of negative nutrients (Table S1). This is with the exception of a higher saturated fat content in cold cereals that met the simple (+53%), dual (+37%) and modified (+38%) ratios, and a higher total sugars (+18%) content in the cereal and fruit bars that met the modified ratio.

#### 3.2.2. Comparison of the Nutritional Composition between Carbohydrate-Based Packaged Beverages

An assessment from Mintel Australia showed that RTD beverages that passed the simple ratio had a lower energy (−20%), total sugars (−40%), and free sugars content (−41%), as well as a greater dietary fibre (+295%) and protein (+51%) content compared to products that failed (Table 3). However, they also had a higher sodium content (+167%).

The analysis of RTD beverages from Mintel Asia found that products that met the simple and dual ratio had a lower total energy (−13% and −19%, respectively), total sugar (−28% and −52%, respectively) and free sugar (−23% and −52%, respectively) content, as well as a greater dietary fibre (+855% and +711%, respectively) content. In addition, RTD beverages that passed the simple ratio also had a lower fat (−26%) and saturated fat (−37%) content. However, RTD beverages that passed the dual ratio had a lower protein (+26%) content (Table 4). 

The analysis of powdered beverages from Mintel Asia found that products that passed any metric had a lower total sugar (−46%, −80% and −86%, respectively) and free sugar (−44%, −79% and −86%, respectively) content, as well as a greater dietary fibre (+347%, +305% and +261%, respectively) and protein (+22%, +28% and +24%, respectively) content. Powdered beverages that passed the simple ratio also had a lower energy (−2%) and sodium (−17%) content. However, powdered beverages that passed the dual and modified ratio had a higher saturated fat content (+23% and +24%, respectively) (Table 4). 

#### 3.2.3. Evaluation of Products from Mintel Database against Nutrient Profiling Models

**Figure 4:**
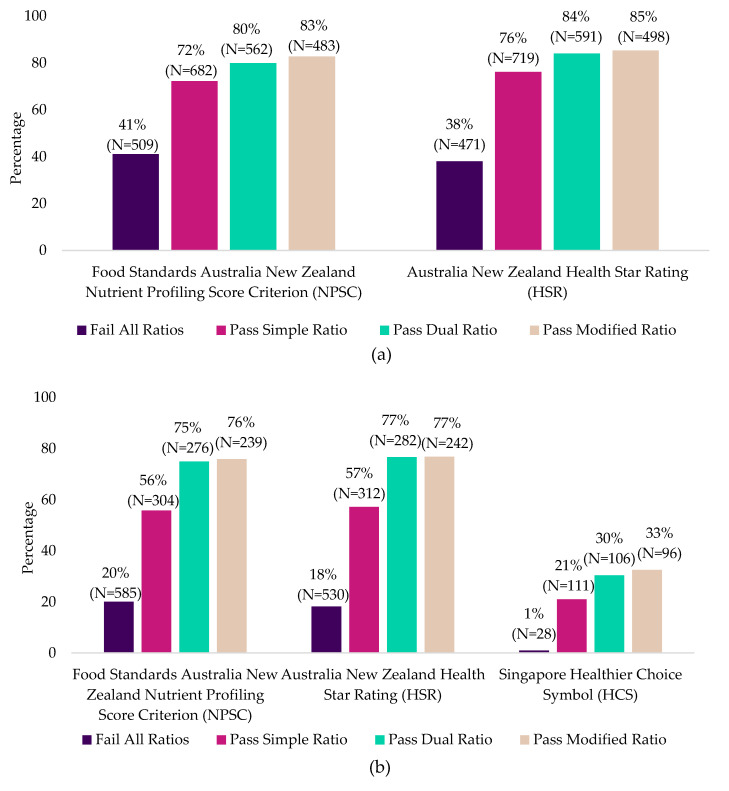
Percentage of carbohydrate-based packaged foods from (**a**) Mintel Australia and (**b**) Mintel Asia that passed or failed the three metrics and met the respective national nutrient profiling models as a nutritious product.

In Mintel Australia, a greater proportion of foods that passed any carbohydrate metric also met the NPSC and HSR as a nutritious food (72–85%) compared to foods that did not (38–41%) (Figure 4a). Similarly, in Mintel Asia, a higher proportion of foods that passed any carbohydrate metric also met the NPSC, HSR and HCS as a nutritious food (21–77%) compared to foods that did not (1–20%) (Figure 4b).

**Figure 5:**
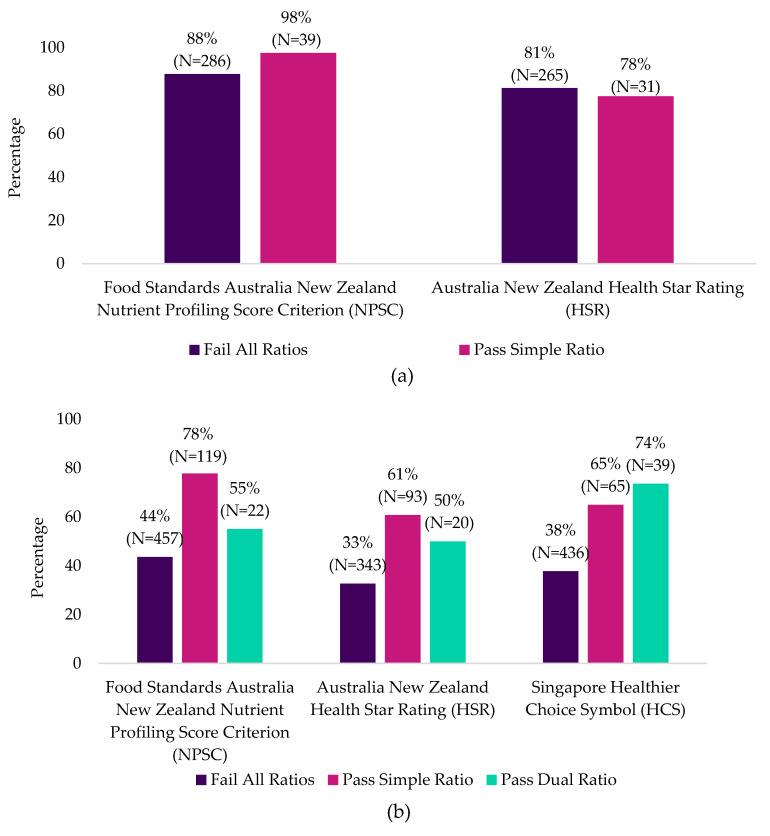

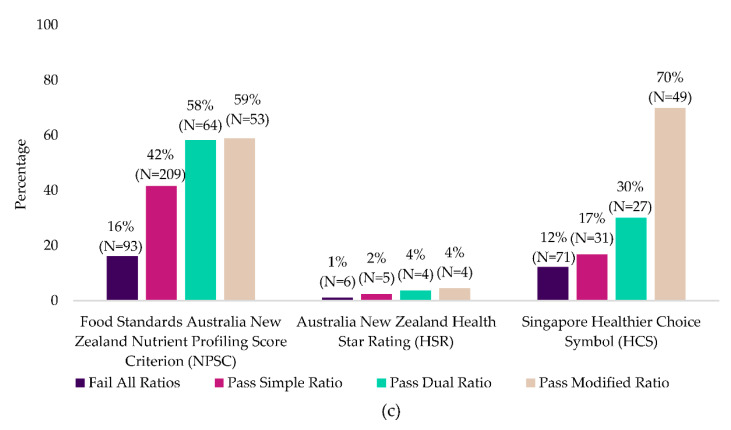
Percentage of carbohydrate-based (**a**) RTD beverages from Mintel Australia (**b**) RTD beverages from Mintel Asia and (**c**) powdered beverages from Mintel Asia that passed or failed the three metrics and met the respective national nutrient product models as a nutritious product.

When beverages were compared against the national nutrient profiling models, there were mixed results. More RTD beverages from Mintel Australia that passed the simple ratio also passed the NPSC criteria (98%), compared to products that failed (88%). However, fewer RTD beverages that passed the simple ratio passed the HSR criteria (78%), compared to products that failed the ratio (81%) (Figure 5a). RTD beverages from Mintel Asia that met any metric were more likely to meet the criteria for the three nutrient profiling models as a nutritious product (50–78%), compared to products that failed (33–44%). Similarly, powdered beverages from Mintel Asia that passed the three metrics were more likely to meet the criteria of the three nutrient profiling models as a nutritious product (2–70%) compared to products that failed all metrics (1–16%) (Figure 5b,c).

### 3.3. Proportion of Whole Grain Product Choices and Their Association with the Carbohydrate Metrics

There was a greater proportion of whole grain food choices from Mintel Australia (31.6%) compared to Mintel Asia (18.8%). There were very few whole grain beverage choices in both Mintel Australia (1.6%) and Mintel Asia (3.7%) (Table S2).

When foods from the Mintel Database were analysed, there was a significant association between adherence to the three metrics and availability of whole grain product variants (*p* < 0.001) (Table S3). When beverages from the Mintel Database were analysed, there was also a significant association between adherence to the simple and dual metrics and the availability of whole grain product variants (*p* < 0.001). However, this was no longer observed with the modified ratio (*p* = 0.070) (Table S4).

### 3.4. Comparison of Data from AFCD-1 and Mintel Australia

A comparison of products sold in Australia between the two databases showed similar results, whereby breads (unfilled), cold cereal and hot cereal had a high adherence and savoury biscuits had a moderate adherence to all of the metrics (Figure 2a,b). Several differences were observed, including cereal and fruit bars to be of high adherence to the simple ratio, and moderate adherence to the dual and modified ratios in Mintel Australia but not in AFCD-1. This is due to the absence of several types of cereal and fruit bars from the AFCD-1 (e.g., cold press bars, meal replacement bars). Additionally, instant pasta and rice had a moderate adherence to all three metrics in Mintel Australia but not in AFCD-1, potentially because the product sampling for AFCD-1 was older.

Figure S1 shows the mean free sugars content of products within a sub-category from Mintel Australia versus the free sugars content of the same sub-category from AFCD-1. The two-tailed t-test conducted showed that there was no significant difference between the free sugars content imputed for the Mintel Database and the free sugars content of the respective product sub-categories in the AFCD-1 (*p* = 0.33). The NSE coefficient of the imputation was analysed to be 0.83. These results verify the concurrency of the free sugars content used in the analysis of products from both databases.

## 4. Discussion

In this study, the adherence of carbohydrate-based packaged foods and beverages to three variations of the carbohydrate metrics characterised by ratios of total carbohydrates, dietary fibre and/or free sugars was examined. Products that met the metrics were generally of a higher nutritional quality and this applied to all countries. Australia had the highest overall number of products meeting all metrics, with twice as many products compared to the Southeast Asian countries. Foods that were predominantly cereal-based had the highest adherence to all metrics, whilst beverages had a relatively low adherence to all metrics compared to foods. 

Previous studies point to the fact that the carbohydrate metrics reflected by similar ratios of total carbohydrates, dietary fibre and/or free sugars may be associated with multiple benefits, as they may help identify more nutritious products [27,28], or improve diet quality [23], thus contributing to chronic disease reduction when calculated on a diet level [6,19]. Findings of the current analysis are consistent with these studies, showing that packaged foods from Southeast Asia and Australia that met the carbohydrate metrics reported a higher nutritional quality, having higher levels of health-promoting nutrients (e.g., dietary fibre, protein, various vitamins and minerals) and lower levels of nutrients of public health concern (e.g., calories, free sugars, trans fat, cholesterol). This is reinforced by the metrics’ alignment with the various national nutrient profiling models to identify nutritious foods and beverages. 

Cereal-based foods, which are more likely to contain whole grains or cereal fibres, had the highest adherence to the metrics. In addition to being a good source of dietary fibre, whole grains are also rich in minerals, vitamins, antioxidants, phytochemicals and other micronutrients that have been linked to disease prevention [40,41], alluding to the higher nutrient density of foods that passed the metrics. However, the presence of whole grains or whole grain communication on a product alone does not necessarily reflect a superior nutritional quality as identified by Mozaffarian et al., who found a higher energy and sugar content in these products in the USA [17]. In some countries (e.g., Singapore), whole grain claims can only be made on the packaging if nutrients of public health concern (e.g., sugar or saturated fat) are within a stipulated threshold [39], ensuring the product’s overall nutritional quality. This discrepancy in results highlights the value of specifying other important nutrient criteria to support content claims such as "high in whole grains".

There were several concessions and interesting findings to the conclusion that foods meeting the metrics were of a higher nutritional quality. In the AFCD-1, foods that passed the metrics had a lower Vitamin A (retinol equivalent) and B12 content. This is likely because cereal-based products in Australia (e.g., cold cereals) are not fortified with these vitamins [42], and the range of product categories that passed the metrics also had a lower content of animal-derived (e.g., liver, fish oils) and carotenoids-rich (e.g., carrots, tomatoes) ingredients, which are rich sources of these vitamins [43]. In the assessment of the Mintel Database, there was occasionally a higher total fat content found in several food categories that passed the metrics. This was due to differences in macronutrient proportion, with products passing the metrics containing fewer total carbohydrates, but more protein and fat in general. While a modest increase in protein content can be nutritionally beneficial [44,45,46], it is important to ensure that the higher fat content does not translate to a greater saturated fat, trans fat, cholesterol or energy content as excessive consumption is associated with metabolic syndrome [47]. Nevertheless, such an occurrence was rarely observed in this study or the analysis of products in the USA [27].

Beverages had a relatively low adherence to all metrics compared to solid foods and were a key category responsible for the steep drop in adherence to the dual and modified metrics when free sugars were taken into consideration. This further highlights previous findings that beverages are a potential source of added and/or free sugars intake in Australia and Southeast Asia [48,49,50,51]. The low adherence to the ratio is specifically due to the intrinsic nature of beverages, causing formulation challenges by removing sugars and incorporating fibres. Most carbohydrates need to be solubilised for the beverage to be palatable and sugars are the most soluble form of carbohydrates [52]. Product formulation is further complicated when dietary fibres are taken into consideration as the former brings in solubility, viscosity and stability challenges [53].

Despite such technical challenges, the simple ratio was able to select nutritionally superior beverages in Australia and Southeast Asia, though it is less clear if the dual and modified ratios selected products of a higher nutritional quality. Beverages that passed the dual and modified ratios were more likely to be low or no sugar beverage variants. Recent governmental and non-governmental pressure on food manufacturers to reduce sugar in beverages may have prompted the growth of such products in the market [54,55,56]. In low or no sugar variants, soluble fibres (e.g., inulin, resistant dextrins) are commonly used in place of sugar to help compensate for the loss of bulk, mouthfeel and stability brought to beverages by sugar [52]. Unlike whole grains, such isolated or synthetic fibre-based ingredients do not bring an added nutritional value beyond its dietary fibre content. Hence, food manufacturers can consider using cereal processing methods (e.g., extrusion, fermentation) to improve formulation of high cereal-fibre beverages [57,58,59,60,61]. Such technologies may improve the palatability of high fibre cereals (e.g., cereal bran or whole grain) in beverages, yet retain the ingredient’s fibre integrity and nutrient density. Given the product category limitations today, from a practical perspective, the simple ratio may be the most relevant metric for identifying beverages with a higher nutritional quality.

These findings are important as studies have reported an increase in consumption of packaged foods and beverages in these countries [25,62,63,64]. Such products have been highlighted to be responsible for the reduced fibre intake and increased sugar, saturated fat and sodium consumption [62,65]. In Southeast Asia, sweetened beverages were identified to be the primary malefactor of sugar intake in the Philippines and Thailand, whilst dried processed foods (e.g., instant noodles) were the main contributors of negative nutrients in Malaysia and Singapore [66]. In Australia, packaged foods and beverages made up 65% of total energy intake and were the major source of free sugar intake [67]. 

There were noteworthy differences observed between countries. Twice the proportion of carbohydrate-based products in Australia met the three metrics compared to the Southeast Asian countries assessed. A possible explanation for this may be the more mature nutrition education program and food labelling regulation in Australia compared to Southeast Asia, allowing both the food industry and population to adapt to healthier foods and the recommendations. For instance, with the exception of Singapore, a front-of-pack labelling system for consumers to identify nutritious variants of products was implemented much earlier in Australia (the HSR scheme) compared to the Southeast Asian countries, which implemented variations of healthier choice schemes only recently [68,69,70,71,72]. Strong governmental and non-governmental advocacy against discretionary food have also compelled food manufacturers to accelerate reformulation of healthier alternatives in Australia [16,73,74,75,76]. Notwithstanding this hypothesis, the key contributing factor likely to account for the large disproportion between these two culturally different regions is the lower availability of whole grain product choices in these Asian countries compared to Australia. This is potentially because whole grain foods are not part of the local eating culture [77], leading to a lower whole grain consumption in Southeast Asia [78,79,80,81,82]. Recent health promotion efforts to increase awareness of the nutritional value of whole grains may increase its intake in these Asian countries in the coming years [78]. Fewer beverages in Australia also passed the dual and modified ratios compared to beverages in Southeast Asia, potentially due to newly instated sugar tax in Thailand, the Philippines and, more recently, Malaysia, which is mostly targeted at beverages [54,55]. It would be of public health interest to monitor the evolution of the overall nutritional quality of these products to validate the effectiveness of these measures to improve the holistic nutritional profile. 

As introduced, there are other indices of carbohydrate quality used by the public health sector, scientific community and food industry. The benefits of the carbohydrate metrics over whole grain claims were addressed above, and common single nutrient content claims such as “high in fibre” or “low in sugar” share a similar limitation whereby a holistic understanding of a product’s nutritional quality might be overlooked [83,84,85]. The GI and glycaemic load (GL) system is one of the more notable measures of carbohydrate quality. When comparing the carbohydrate metrics against this as a measure of overall nutritional quality, both systems were found to have their merits and shortcomings. The GI and GL of diets are predictive of T2DM and heart disease risk [10,11,12,86]. However, Goff and team found that low GI diets were only effective against cardiovascular disease when it had sufficient fibre [87], the principal criteria of the carbohydrate metrics. As described earlier, there is a limited understanding and use of the GI and GL system due to its complexity [13], and the inter-subject and even intra-subject day-to-day variability observed during clinical testing limits its reliability [88,89]. This has led to a lack of consensus amongst the scientific community on its practical use as a measure of nutritional quality [7,11,88,89,90] and inconsistent global legislative advice of its application for product communication [13,91,92,93]. Nevertheless, future studies are required to compare the effectiveness of the carbohydrate metrics, whole grain content and GI as measures of nutritional quality and a means of nutrition communication to evaluate how the different approaches can be complementary to each other.

An advantage of the carbohydrate metric is that it provides a straightforward means to assess nutritional quality based on nutritional content. However, labelling regulations across countries are an important consideration for its implementation. Carbohydrates are declared as available carbohydrates (e.g., Australia) in some countries and the declaration of added/free sugars is not compulsory in most countries, including the countries assessed in this paper [94,95]. This may add confusion to consumer understanding of the carbohydrate metrics. On the other hand, the metric does present an opportunity to increase awareness of the importance of these nutrients, helping consumers select carbohydrate-rich products of a higher nutritional quality, as diets with a high dietary fibre, high whole grain and low free sugar intake have all been associated with a reduction in risk to several chronic diseases [7,21,22,79]. A potential limitation of the metric is its inability to indisputably account for the amount of readily digestible starches in a product [27]. High intakes of refined carbohydrates contribute to metabolic syndrome and an increased risk to its associated diseases [86,96]. However, the fact that the metric has set a minimum for the dietary fibre content in relation to the total carbohydrates content allows it to partially control for this, as the simple ratio criteria effectively selects for less refined whole grain variants of starch-based foods [17].

This study has several limitations and strengths. Firstly, it focuses on products sold in a country, but a direct analysis against consumption levels of these products or diet-disease data could not be carried out due to data unavailability. Further analyses linking product nutritional quality with dietary intake at a population level may provide a clearer picture of the real impact on diet quality. Secondly, both databases employed have their limitations. Data from AFCD-1 are aggregated in sub-categories obtained from a sampling of common Australian products, of which several had not been updated in recent years. In the Mintel Database, the free sugars content employed for the analysis had to be imputed from the declared total sugars content. This study addressed some of the limitations by complementing the strengths of each database. The Mintel Database reflected recent products out in the market today and provided granularity for product assessment. Its free sugars imputation was validated against the AFCD-1. Results between AFCD-1 and Mintel were comparable, and the overall findings of this analysis parallel the previous studies cited [23,27,28]. The study also provides a valuable comparison of carbohydrate-based products in two culturally diverse regions. These findings have public health implications and show that both government (e.g., claims and product labelling regulation) and non-governmental advocacy for nutritious products have positive implications on products in the market and, thus, potentially population level nutritional intake. 

Whilst the findings of the previous studies [23,27,28] and this analysis show that products that meet the carbohydrate metrics have an improved nutritional quality, whether the consumption of products or diets meeting the dual and modified ratios contribute to improved health outcomes remains to be validated. Therefore, future research from epidemiological and clinical studies to correlate dietary data with disease risk can help strengthen the integrity of the carbohydrate metrics. If validated, the carbohydrate metrics may be translated for public health use such as through novel front-of-pack labels or as guidelines for food manufacturers to develop healthier carbohydrate-based foods.

## 5. Conclusions

The carbohydrate metric is a straightforward indicator of product nutritional quality and is relevant to Asian and Australian packaged foods and beverages. In all investigated countries, it allowed identification of carbohydrate-based products rich in whole grains, dietary fibre and other health-promoting nutrients, but lower in energy density, free sugars and other nutrients of public health concern. In solid foods, all three metrics were good indicators of healthier carbohydrate-based packaged foods, whilst in beverages, due to the nature of the product category, the simple ratio was the best metric for selecting products of a higher nutritional density and alignment with national nutrient profiling models. The carbohydrate metrics can be used by consumers to select more nutritious carbohydrate-rich products. Findings from this study may also contribute to product nutritional guidelines for governments and guide food manufacturers on product reformulation.

## Figures and Tables

**Table 1:** Packaged food and beverage categories in the scope of this analysis and their descriptions.

Product Categories	Description
Breads (Unfilled)	Breads with no filling or topping (e.g., bread loaves, naan, pita)
Breads (Filled/Toppings)	Breads with filling (e.g., cream) or toppings (e.g., raisins)
Cold Cereals	Ready-to-eat breakfast cereals which are typically consumed cold with milk or milk substitutes (e.g., muesli, corn flakes)
Hot Cereals	Solid or semi-solid cereal products that are intended to be consumed hot (e.g., oatmeal, porridges)
Cereal and Fruit Bars	Snack bars that are cereal or fruit-based
Processed Grain-based Staples	Staples which have been processed (pastas and noodles)
Savoury Biscuits	Biscuits with a savoury flavour
Sweet Biscuits	Biscuits with a sweet flavour (e.g., cookies, sandwich biscuits)
Sweet Pastries and Desserts	Ready-to-eat desserts (e.g., cakes, muffins, pies, tarts, scones, puddings, pastries)
Instant Noodles	Dried noodles with added flavours for a soup or sauce base
Instant Pasta and Rice	Dried or chilled pasta or rice ready meals with added flavours for a sauce base
Pizza	Chilled or frozen pizza with toppings
All Foods	Aggregation of all food categories assessed
Cereal Mixes	Cereal-based (e.g., oat, rice, barley, corn) beverages, often containing solid cereal flakes/bits
Flavoured Milk/Malt Beverages	Flavoured-milks (e.g., chocolate milk), powdered concentrates for flavoured milks (e.g., hot chocolate) and flavoured malt-based beverages which also contain dairy
Juices	Beverages that are >95% fruit or vegetable juice, either freshly squeezed or from concentrate
Flavoured Juice	Non-carbonated fruit or vegetable flavoured juices, excluding flower/herbal drinks
Plant-based Milks	Dairy-based alternative drinks
Tea and Coffee Mixes	Tea and coffee-based beverages with more than one ingredient (e.g., sugar, creamer, milk)
All Beverages	Aggregation of all beverage categories assessed
All Products Assessed	Aggregation of all food and beverage categories assessed

**Table 2:** Comparison of mean nutrient composition of carbohydrate-based packaged food sub-categories from AFCD-1 that passed or failed all of the three carbohydrate metrics.

Nutrient	Unit	Mean		*p* Value
		Pass	Fail	
Percentage of sub-categories (*n* = 86)		22	78	
Energy	kcal	318.9	333.3	0.399
Protein	g	10.4	7.8	<0.001
Total Fat	g	4.7	9.0	<0.001
Saturated fat	g	0.9	3.7	<0.001
Trans fat	mg	20.9	176.0	<0.001
Cholesterol	mg	0.0	22.2	<0.001
Total Carbohydrates	g	63.3	56.9	0.080
Available Carbohydrates	g	53.1	54.1	0.759
Dietary Fibre	g	10.2	2.8	<0.001
Total Sugars	g	7.0	15.4	0.001
Free Sugars	g	2.7	12.2	<0.001
Sodium	mg	279.1	385.2	0.052
Calcium	mg	59.6	74.1	0.411
Iron	mg	4.5	2.0	0.010
Iodine	ug	31.1	17.5	0.061
Magnesium	mg	97.0	30.3	<0.001
Potassium	mg	337.4	168.3	0.001
Zinc	mg	2.1	1.0	0.005
Selenium	ug	11.8	8.2	0.011
Vitamin A (Retinol Equivalent)	ug	0.8	37.5	<0.001
Vitamin E	mg	0.9	0.9	0.694
Vitamin B1	mg	0.7	0.3	0.014
Vitamin B2	mg	0.3	0.2	0.377
Vitamin B3	mg	4.4	1.9	0.014
Pyridoxine B6	mg	0.2	0.1	0.179
Vitamin B9 (Dietary Folate Equivalent)	ug	231.3	109.5	0.037
Vitamin B12	ug	0.0	0.2	0.013

**Table 3:** Comparison of mean nutrient composition of carbohydrate-based packaged food and beverage products from Mintel Australia that passed or failed the three carbohydrate metrics.

		Simple Ratio				Dual Ratio		Modified Ratio		
Nutrient	Unit	Mean		*p* Value	Mean		*p* Value	Mean		*p* Value
		Pass	Fail		Pass	Fail		Pass	Fail	
Foods (*n* = 2190)										
Percentage of Products		43	57		32	68		27	73	
Energy	Kcal	364.8	368.0	0.394	355.7	371.7	<0.001	349.0	373.0	<0.001
Dietary Fibre	g	10.2	3.4	<0.001	10.9	4.1	<0.001	10.6	4.7	<0.001
Total Sugars	g	16.8	17.2	0.566	14.1	18.4	<0.001	13.5	18.3	<0.001
Free Sugars	g	9.9	14.9	<0.001	5.6	16.1	<0.001	3.3	16.2	<0.001
Protein	g	10.5	7.7	<0.001	10.8	8.0	<0.001	10.7	8.3	<0.001
Total Fat	g	10.3	9.5	0.005	9.5	10.0	0.114	8.9	10.1	<0.001
Saturated Fat	g	2.8	3.8	<0.001	2.5	3.8	<0.001	2.3	3.8	<0.001
Sodium	mg	167.0	866.0	0.216	170.0	750.0	0.220	174.0	706.0	0.225
Ready-to-Drink (RTD) Beverages (*n* = 374)										
Percentage of Products		11	89							
Energy	Kcal	34.2	42.8	0.005	No analysis was conducted as only 6 products passed this metric			No analysis was conducted as no products passed this metric		
Dietary Fibre	g	1.2	0.3	<0.001						
Total Sugars	g	4.9	8.3	<0.001						
Free Sugars	g	4.8	8.1	<0.001						
Protein	g	0.8	0.6	0.032						
Total Fat	g	0.5	0.4	0.327						
Saturated Fat	g	0.2	0.2	0.673						
Sodium	mg	45.9	17.2	0.038						

**Table 4:** Comparison of mean nutrient composition of carbohydrate-based packaged food and beverage products from Mintel Asia that passed or failed the three carbohydrate metrics.

		Simple Ratio				Dual Ratio		Modified Ratio		
Nutrient	Unit	Mean		*p* Value	Mean		*p* Value	Mean		*p* Value
		Pass	Fail		Pass	Fail		Pass	Fail	
Foods (*n* = 3583)										
Percentage of Products		16	84		11	89		9	91	
Energy	Kcal	374.1	403.1	<0.001	359.2	403.2	<0.001	357.0	402.7	<0.001
Dietary Fibre	g	9.7	2.8	<0.001	10.4	3.1	<0.001	10.1	3.2	<0.001
Total Sugars	g	14.1	20.9	<0.001	8.5	21.2	<0.001	6.9	21.2	<0.001
Free Sugars	g	11.4	20.0	<0.001	4.9	20.3	<0.001	2.7	20.2	<0.001
Protein	g	9.5	7.5	<0.001	10.2	7.5	<0.001	10.5	7.5	<0.001
Total Fat	g	10.5	12.2	<0.001	8.9	12.3	<0.001	8.7	12.2	<0.001
Saturated Fat	g	4.0	6.0	<0.001	2.8	6.0	<0.001	2.8	5.9	<0.001
Sodium	mg	295.0	1219.0	<0.001	295.0	1167.0	<0.001	315.0	1150.0	<0.001
Ready-to-Drink (RTD) Beverages (*n* = 1397)										
Percentage of Products		13	87		4	96		No analysis was conducted as only 13 products passed this metric		
Energy	Kcal	43.7	50.1	<0.001	40.1	49.6	0.002			
Dietary Fibre	g	2.1	0.2	<0.001	3.0	0.4	<0.001			
Total Sugars	g	6.3	8.7	<0.001	4.1	8.6	<0.001			
Free Sugars	g	6.1	7.9	<0.001	3.8	7.9	<0.001			
Protein	g	0.8	0.9	0.113	0.7	0.9	0.025			
Total Fat	g	0.5	0.7	0.007	0.6	0.7	0.365			
Saturated Fat	g	0.2	0.3	0.001	0.3	0.3	0.858			
Sodium	mg	230.0	135.0	0.646	36.7	153.0	0.057			
Powdered Beverages (*n* = 833)										
Percentage of Products		27	73		14	86		11	89	
Energy	Kcal	413.0	423.3	0.031	411.9	422.0	<0.001	413.3	421.5	0.227
Dietary Fibre	g	13.2	3.0	<0.001	16.1	4.0	<0.001	15.9	4.4	<0.001
Total Sugars	g	25.3	47.1	<0.001	9.5	46.5	<0.001	6.2	45.7	<0.001
Free Sugars	g	24.5	43.5	<0.001	8.9	43.3	<0.001	6.0	42.6	<0.001
Protein	g	7.5	6.1	0.001	8.0	6.2	<0.001	7.8	6.3	0.018
Total Fat	g	11.2	10.1	0.039	12.0	10.1	<0.001	12.3	10.2	0.009
Saturated Fat	g	9.0	8.0	0.107	9.9	8.0	0.023	10.0	8.1	0.019
Sodium	mg	161.0	194.0	0.011	172.0	187.0	0.466	134.0	191.0	<0.001

## Supplementary Materials

The following are available online at https://www.mdpi.com/2072-6643/12/9/2771/s1, Table S1. Comparison of mean nutrient composition of hot cereals, cold cereals, cereal and fruit bars, and breads (unfilled) from the Mintel Database which pass or fail the three carbohydrate metrics, Table S2. Proportion of packaged carbohydrate-based foods and beverages which have whole grain claims and/or communication, Table S3. Association between carbohydrate metrics and whole grain claims and/or communication for foods assessed from the Mintel Database, Table S4. Association between carbohydrate metrics and whole grain claims and/or communication for beverages assessed from the Mintel Database, Figure S1. Mean free sugars content of products within a sub-category from Mintel Australia versus the free sugars content of the same sub-category from AFCD-1. Each point represents a product sub-category. The mean free sugars content of 1565 products which belonged to 69 sub-categories in AFCD-1 were tabulated.
