# Supplementary material for: An Assessment of Three Carbohydrate Metrics of Nutritional Quality for Packaged Foods and Beverages in Australia and Southeast Asia
Source: Nutrients. 2020 Sep 11;12(9):2771. doi: 10.3390/nu12092771 (PMC7551443; doi:10.3390/nu12092771)
Supplement: Supplementary file 1 [file nutrients-12-02771-s001.pdf]

Table S1. Comparison of mean nutrient composition of hot cereals, cold cereals, cereal and fruit bars, and breads (unfilled) from the Mintel Database which pass or fail the three carbohydrate metrics

Nutrient	Unit	Simple Ratio			Dual Ratio			Modified Ratio		
		Mean		P	Mean		P	Mean		P
		Pass	Fail	Value	Pass	Fail	Value	Pass	Fail	Value
**Hot Cereals (N=279)**										
**Percentage of Products**		75	25		66	34		62	38	
**Energy**	Kcal	373.8	387.9	0.038	376.9	378.1	0.873	375.8	379.7	0.566
**Total Carbohydrates**	g	65.4	73.7	<0.001	65.5	71.3	<0.001	65.5	70.6	<0.001
**Dietary Fibre**	g	10.5	5.7	<0.001	8.3	6.7	<0.001	10.9	6.7	<0.001
**Total Sugars**	g	5.5	20.3	<0.001	3.5	20.2	<0.001	2.4	19.9	<0.001
**Free Sugars**	g	4.6	19.8	<0.001	2.5	19.8	<0.001	1.3	19.6	<0.001
**Protein**	g	12.2	9.9	<0.001	12.6	9.8	<0.001	12.7	10.0	<0.001
**xTotal Fat**	g	8.1	6.7	<0.001	8.3	6.7	<0.001	8.2	7.0	<0.001
**Saturated Fat**	g	1.7	2.3	0.073	1.7	2.0	0.210	1.6	2.2	0.013
**Sodium**	mg	67.0	343.0	<0.001	65.0	276.0	<0.001	65.0	251.0	<0.001
**Cold Cereals (N=933)**										
**Percentage of Products**		46	54		37	63		31	69	
**Energy**	Kcal	386.5	389.4	0.249	382.2	391.5	0.001	381.1	391.2	0.001
**Total Carbohydrates**	g	70.2	79.9	<0.001	69.7	78.7	<0.001	69.5	78.1	<0.001
**Dietary Fibre**	g	11.1	4.7	<0.001	11.8	5.3	<0.001	11.5	5.9	<0.001
**Total Sugars**	g	16.9	25.7	<0.001	15.4	25.3	<0.001	15.0	24.6	<0.001
**Free Sugars**	g	10.8	23.5	<0.001	7.7	23.4	<0.001	6.0	22.9	<0.001
**Protein**	g	10.3	8.0	<0.001	10.6	8.2	<0.001	10.5	8.4	<0.001
**Total Fat**	g	9.2	4.8	<0.001	9.0	5.6	<0.001	8.9	5.9	<0.001
**Saturated Fat**	g	2.1	1.3	<0.001	2.0	1.5	<0.001	2.1	1.5	<0.001
**Sodium**	mg	139.0	293.0	<0.001	130.0	276.0	<0.001	124.0	266.0	<0.001
**Cereal & Fruit Bars (N=680)**										
**Percentage of Products**		56	44		31	69		19	81	
**Energy**	Kcal	385.4	398.1	0.002	381.8	395.1	<0.001	379.7	393.6	0.006
**Total Carbohydrates**	g	62.9	68	<0.001	62.1	66.5	<0.001	60.9	66.1	<0.001
**Dietary Fibre**	g	10.2	4.0	<0.001	11.9	5.4	<0.001	11.5	6.5	<0.001
**Total Sugars**	g	27.5	31.1	<0.001	27.4	29.9	<0.001	33.2	28.2	0.001
**Free Sugars**	g	15.2	26.4	<0.001	8.4	25.4	<0.001	2.4	24.2	<0.001
**Protein**	g	10.2	6.9	<0.001	10.6	7.9	<0.001	10.4	8.3	0.002
**Total Fat**	g	13.3	11.8	<0.001	13.3	12.3	<0.001	14.0	12.3	0.002
**Saturated Fat**	g	4.4	5.2	0.003	4.1	5.0	0.001	4.2	4.9	0.056
**Sodium**	mg	110.0	168.0	<0.001	95.0	153.0	<0.001	66.0	151.0	<0.001
**Breads (Unfilled) (N=306)**										
**Percentage of Products**		34	66							
**Energy**	Kcal	252.2	290.0	<0.001						
**Total Carbohydrates**	g	46.2	51.4	<0.001						
**Dietary Fibre**	g	7.5	2.9	<0.001						
**Total Sugars**	g	3.1	5.0	<0.001						
**Free Sugars**	g	0.0	0.0	-	All Breads (Unfilled) which passed for simple ratio also passed for the dual and modified ratios					
**Protein**	g	9.2	8.7	0.144						
**Total Fat**	g	4.3	5.9	<0.001						
**Saturated Fat**	g	1.5	2.4	<0.001						
**Sodium**	mg	391.0	434.0	0.011						

Table S2. Proportion of packaged carbohydrate-based foods and beverages which have whole grain claims and/or communication

Region	Percentage of Foods with Whole Grain Positioning	Percentage of Beverages with Whole Grain Positioning
**Australia**	31.6% (N=692)	1.6% (N=6)
**Southeast Asia**	18.8% (N=672)	3.7% (N=82)

Table S3. Association between carbohydrate metrics and whole grain claims and/or communication for foods assessed from the Mintel Database

Number of products	Simple Ratio		Dual Ratio		Modified Ratio	
	Pass	Fail	Pass	Fail	Pass	Fail
**Whole Grain Claim**	731	633	540	824	438	926
**No Whole Grain Claim**	781	3628	549	3860	478	3931
**Pearson Chi-Square**	693.7		501.3		353.0	
**P-Value**	<0.001		<0.001		<0.001	

Table S4. Association between carbohydrate metrics and whole grain claims and/or communication for beverages assessed from the Mintel Database

Number of products	Simple Ratio		Dual Ratio		Modified Ratio	
	Pass	Fail	Pass	Fail	Pass	Fail
**Whole Grain Claim**	29	59	18	70	7	81
**No Whole Grain Claim**	424	2105	163	2366	102	2427
**Pearson Chi-Square**	15.6		25.9		3.3	
**P-Value**	<0.001		<0.001		0.070	

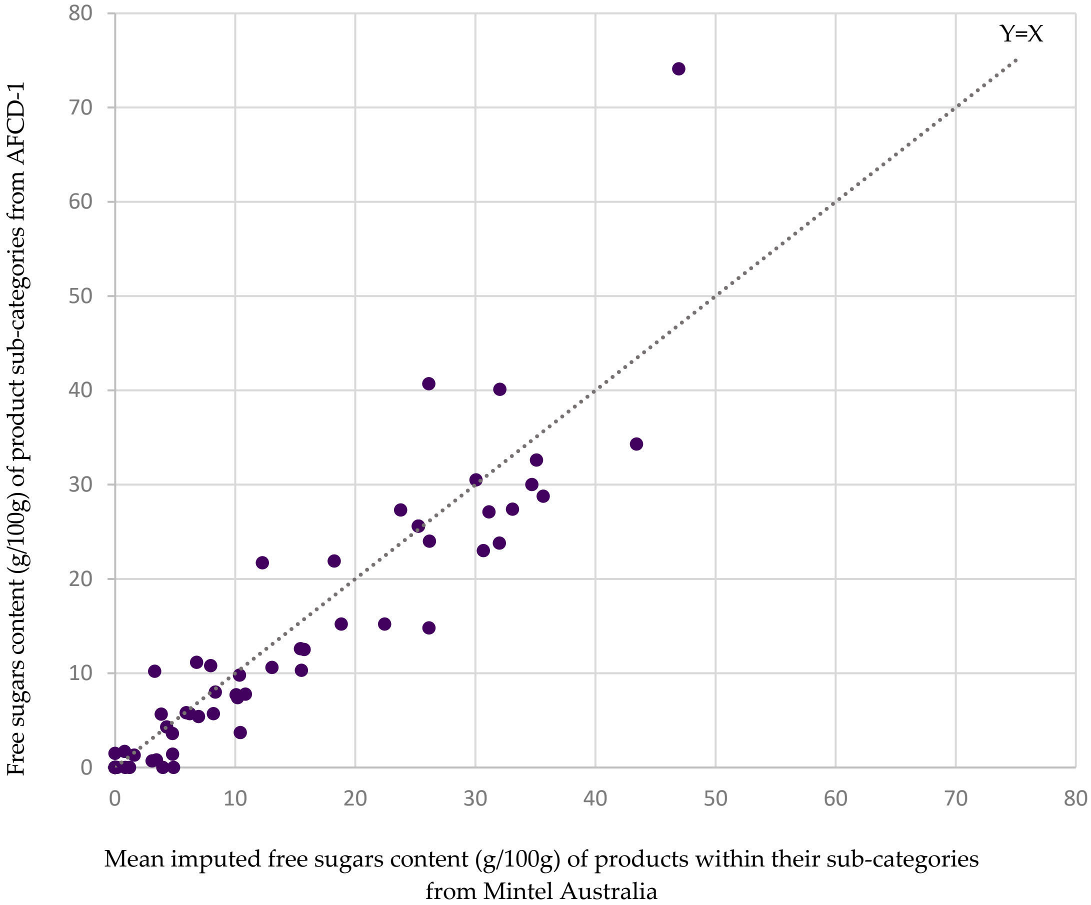

Figure S1. Mean free sugars content of products within a sub-category from Mintel Australia versus the free sugars content of the same sub-category from AFCD-1. Each point represents a product sub-category. The mean free sugars content of 1565 products which belonged to 69 sub-categories in AFCD-1 were tabulated.
